# BDNF mediates improvements in executive function following a 1-year exercise intervention

**DOI:** 10.3389/fnhum.2014.00985

**Published:** 2014-12-11

**Authors:** Regina L. Leckie, Lauren E. Oberlin, Michelle W. Voss, Ruchika S. Prakash, Amanda Szabo-Reed, Laura Chaddock-Heyman, Siobhan M. Phillips, Neha P. Gothe, Emily Mailey, Victoria J. Vieira-Potter, Stephen A. Martin, Brandt D. Pence, Mingkuan Lin, Raja Parasuraman, Pamela M. Greenwood, Karl J. Fryxell, Jeffrey A. Woods, Edward McAuley, Arthur F. Kramer, Kirk I. Erickson

**Affiliations:** ^1^Department of Psychology, University of PittsburghPittsburgh, PA, USA; ^2^Center for the Neural Basis of Cognition, University of PittsburghPittsburgh, PA, USA; ^3^Department of Psychology, University of IowaIowa City, IA, USA; ^4^Department of Psychology, Ohio State UniversityColumbus, OH, USA; ^5^Cardiovascular Research Institute, University of Kansas Medical CenterKansas City, KS, USA; ^6^Beckman Institute for Advanced Science and Technology, University of IllinoisChampaign-Urbana, IL, USA; ^7^Department of Preventative Medicine, Northwestern University Medical SchoolChicago, IL, USA; ^8^Department of Kinesiology, Wayne State UniversityDetroit, MI, USA; ^9^Department of Kinesiology, Kansas State UniversityManhattan, KS, USA; ^10^Department of Kinesiology and Community Health, University of IllinoisChampaign-Urbana, IL, USA; ^11^Department of Neuroscience, George Mason UniversityFairfax, VA, USA; ^12^Department of Psychology, George Mason UniversityFairfax, VA, USA; ^13^School of Molecular Biology, George Mason UniversityFairfax, VA, USA; ^14^Center for Neuroscience, University of PittsburghPittsburgh, PA, USA

**Keywords:** BDNF, executive function, aging, exercise, physical activity, cognition, mediation analysis

## Abstract

Executive function declines with age, but engaging in aerobic exercise may attenuate decline. One mechanism by which aerobic exercise may preserve executive function is through the up-regulation of brain-derived neurotropic factor (BDNF), which also declines with age. The present study examined BDNF as a mediator of the effects of a 1-year walking intervention on executive function in 90 older adults (mean age = 66.82). Participants were randomized to a stretching and toning control group or a moderate intensity walking intervention group. BDNF serum levels and performance on a task-switching paradigm were collected at baseline and follow-up. We found that age moderated the effect of intervention group on changes in BDNF levels, with those in the highest age quartile showing the greatest increase in BDNF after 1-year of moderate intensity walking exercise (*p* = 0.036). The mediation analyses revealed that BDNF mediated the effect of the intervention on task-switch accuracy, but did so as a function of age, such that exercise-induced changes in BDNF mediated the effect of exercise on task-switch performance only for individuals over the age of 71. These results demonstrate that both age and BDNF serum levels are important factors to consider when investigating the mechanisms by which exercise interventions influence cognitive outcomes, particularly in elderly populations.

## Introduction

Decline in processing speed, memory, and executive function is a relatively widespread characteristic of aging (Li et al., 2001; Bishop et al., 2010; Silver et al., 2011; El Haj and Allain, 2012; Singh-Manoux et al., 2012; Woodard et al., 2012). Several neural mechanisms thought to support these cognitive processes become particularly vulnerable during the aging process (Burke and Barnes, 2006). For instance, brain-derived neurotrophic factor (BDNF) levels tend to decrease progressively with age (Lommatzsch et al., 2005; Ziegenhorn et al., 2007; Driscoll et al., 2012) and correlate with age-related reductions in hippocampal volume (Erickson et al., 2010a). BDNF facilitates neural repair (Yang et al., 2014), induces long-term potentiation (Diógenes et al., 2011), enhances learning and memory (Pang and Lu, 2004; Bekinschtein et al., 2014), and promotes synaptic plasticity and neurogenesis (Oliff et al., 1998; Lu, 2003; Vaynman et al., 2004). The beneficial effects of BDNF on brain health and cognition may be especially relevant for older adults, who are susceptible to physiological changes that interfere with neural processes and cognitive function. For instance, marked increases in blood pressure and inflammatory load occur in older adulthood, both of which have been independently linked to dementia risk and decline in cognitive function (Schmidt et al., 2002; Singh and Newman, 2011). Thus, there is an increased presence of factors that compromise cognitive function in older adults, with a contemporaneous decrease in known mechanisms that support advantageous brain and cognitive health. Therefore, efforts to increase BDNF, particularly in older aged individuals, could play an important role in the preservation of cognitive function.

Fortunately, exercise may be an effective approach to preserve and improve cognitive function and brain health in late adulthood. For example, randomized clinical trials have found that moderate-intensity exercise improves memory and executive function (Kramer et al., 1999a; Colcombe and Kramer, 2003; Smith et al., 2010) increases prefrontal cortex (Colcombe et al., 2006) and hippocampal volume (Erickson et al., 2011), and influences brain network connectivity (Voss et al., 2010a). Cross-sectional and epidemiological studies have supported these results and demonstrate that higher fitness levels and greater amounts of physical activity are associated with greater gray matter volumes (Erickson et al., 2010b; Weinstein et al., 2012; Benedict et al., 2013), white matter integrity (Gow et al., 2012; Gons et al., 2013; Tian et al., 2014), reduced brain atrophy rates (Gow et al., 2012; Smith et al., 2014), and reduced risk of cognitive impairment and dementia (Podewils et al., 2005; Larson et al., 2006; Sofi et al., 2011). Yet, the mechanisms by which exercise improves or maintains brain health in humans remain poorly understood, but likely include changes in inflammation, insulin resistance, as well as central changes in serotonin, dopamine, or other neurotransmitters.

Increased expression of BDNF may be another mechanism by which exercise positively influences cognitive and brain function. For example, in rodents, exercise increases BDNF expression in the striatum (Marais et al., 2009), hippocampus (Aguiar et al., 2011), and cortex (Neeper et al., 1996) and an exercise-induced up-regulation of BDNF, along with its receptor tyrosine kinase (Li et al., 2008), mediates the effects of exercise on cognition (Gómez-Pinilla et al., 2002; Vaynman et al., 2004; Van Praag et al., 2005; Stranahan et al., 2009; Creer et al., 2010; Bechara and Kelly, 2013). Unfortunately in humans the effect of exercise on serum BDNF is more equivocal (Zoladz and Pilc, 2010). Acute bouts of exercise increase serum BDNF levels, which, in turn, are associated with improvements in cognitive performance (Knaepen et al., 2010; Ströhle et al., 2010; Heyman et al., 2012; Coelho et al., 2013). However, more chronic and longer-term exercise programs have not reliably demonstrated increases in BDNF levels (Baker et al., 2010; Erickson et al., 2011; Ruscheweyh et al., 2011). Despite these discouraging effects of exercise interventions, exercise-induced changes in hippocampal volume (Erickson et al., 2011) and brain connectivity (Voss et al., 2013) were correlated with percent change in serum BDNF levels. These findings suggest that serum BDNF levels may be an important biomarker for brain and cognitive health, but whether BDNF mediates the positive effects of exercise on cognitive performance remains a matter of speculation.

Here, we examine whether exercise-induced changes in serum BDNF mediates the effect of a randomized exercise intervention on executive function. Specifically, we assessed effects on task-switch performance, a cognitive task largely susceptible to age-related shifts in performance speed and accuracy (Salthouse et al., 1998; Kray and Lindenberger, 2000; Cepeda et al., 2001; Jimura and Braver, 2010; Gazes et al., 2012). This task is considered a measure of executive control and is supported primarily by the prefrontal and parietal cortices (Sohn et al., 2000; Braver et al., 2003; Aron et al., 2004; Gold et al., 2010), regions that experience preferential degeneration with advancing age (Sowell et al., 2003; Salat et al., 2005; Driscoll et al., 2009). Because BDNF levels decline with age (Lommatzsch et al., 2005; Burke and Barnes, 2006; Ziegenhorn et al., 2007), and is found in cortical regions that likely support task-switch performance (Podewils et al., 2005; Aguiar et al., 2011; Diógenes et al., 2011), we reasoned that age might be an important moderator of the effects of exercise on BDNF and task-switch performance.

Prior studies examining the effects of exercise on serum BDNF have used age as a covariate rather than modeling age as an interaction term. Yet, several studies and meta-analyses suggest that the positive effects of exercise on cognitive performance may be magnified at older ages (Etnier et al., 2007; Smith et al., 2010) while others have found that age-related losses in gray matter volume and n-acetylaspartate levels were mitigated by higher fitness levels (Colcombe et al., 2003; Erickson et al., 2012). These and other studies (Adlard et al., 2005; Knaepen et al., 2010) suggest that age might be moderating an effect of exercise on serum BDNF levels.

The current study examined the effects of a 12-month randomized exercise intervention on serum BDNF and task-switching performance in older adults. We predicted that exercise-induced changes in serum BDNF would mediate the effects of the exercise intervention on task-switch performance. However, we also predicted that age would moderate the effect of the intervention on BDNF levels, such that the oldest adults in the exercise group would show greater increases in BDNF than their younger counterparts.

## Method

### Participants

One-hundred and seventy-nine participants were randomly assigned to either a walking exercise or stretching-toning control group prior to the start of the intervention. Of the 179 participants, 106 had complete blood data collected at baseline and post-intervention for BDNF assays. Additionally, 13 participants were missing *BDNF* genetic polymorphism data and were excluded from the analysis. We also excluded one participant who was ill at the time of blood draw. Our final sample in BDNF analyses consisted of 92 participants. There were an additional two individuals missing task-switching data (one from each group), so all analyses conducted with the task-switch data used a sample of 90 participants. Excluded participants did not differ in demographic characteristics or baseline fitness level from those included (*p* > 0.05). The sample and results from this intervention have been previously reported, where hippocampal volume (Erickson et al., 2011), brain connectivity (Voss et al., 2010a,b, 2013) white matter integrity and adherence to the intervention (McAuley et al., 2011) were examined. The analyses and results reported in the current study have not been previously examined, nor published.

Community-dwelling older adults were recruited from the local community of Urbana-Champaign, Illinois. Eligible participants had to (*i*) demonstrate strong right handedness (Oldfield, 1971, *ii*) be between the ages of 55 and 80, (*iii*) score ≥51 on the modified Mini-Mental Status Examination (Stern et al., 1987, Singh-Manoux et al., 2012), score <3 on the Geriatric Depression Scale to rule out possible depression (Yesavage and Sheikh, 1986, *v*) have normal color vision, (*vi*) have a corrected visual acuity of at least 20/40, (*vii*) have no history of neurological diseases or infarcts, including Parkinson's disease, Alzheimer's disease, multiple sclerosis, or stroke, (*viii*) have no history of major vasculature problems, including cardiovascular disease or diabetes, (*ix*) obtain consent from their personal physician, and (*x*) sign an informed consent form approved by the University of Illinois. In addition, all participants had to report being currently sedentary, which was defined as having been physically active for 30 min or more no more than two times in the last 6 months. Participants were compensated for their participation. The Institutional Review Board of the University of Illinois approved the study and all participants gave written informed consent prior to participating.

After completion of the initial blood draw, an MRI session (results not included here), and fitness assessment, participants were randomized to the aerobic walking group (*n* = 47) or to the stretching-toning control group (*n* = 45). Demographic information can be found in Table 1.

**Table 1:** **Demographics, fitness, and BDNF**.

**Variable**	**Total sample *N* = 92**	**Walking group *N* = 47**	**Control group *N* = 45**
% Men	35.9%	31.90%	40.00%
Age (years)	66.82 (5.59)	67.23 (5.39)	66.38 (5.83)
**EDUCATION %**			
High School	19.6%	19.1%	20%
Part College/Vocational	30.4%	36.2%	24.4%
Bachelor's Degree	16.3%	12.8%	20%
Master's Degree	21.7%	23.4%	20%
PhD or Equivalent	12%	8.5%	15.6%
VO_2max_(mL/kg)	21.51 (4.68)	21.22 (4.62)	21.82 (4.78)
Pre-intervention BDNF (pg/mL)	21,909.96 (9147.95)	21,736.91 (9768.31)	22,090.69 (8558.71)
Post-intervention BDNF (pg/mL)	22,897.03 (8293.85)	24,067.78 (7814.90)	21,674.24 (8684.70)
BDNF met allele carriers	7	2	5

*Demographic information comparing the two intervention groups: walking intervention, stretching and toning control*.

### Aerobic fitness assessment

Maximal oxygen uptake (VO_2max_) was used as an objective measure of baseline cardiorespiratory fitness. Participants were required to obtain consent from their personal physician before cardiorespiratory fitness testing was conducted. As detailed by Voss et al. (2010a) assessment of cardiorespiratory fitness was determined using graded maximum exercise testing on a motor-driven treadmill with continuous monitoring of respiration, heart rate, and blood pressure by a cardiologist and nurse (Voss et al., 2010a, 2013). During the assessment, subjects walked at a speed slightly faster than their normal walking pace with increasing graded increments of 2% every 2 min. Oxygen uptake was measured at 30-s intervals until a max VO_2_ was attained or to the point of test termination due to exhaustion. VO_2max_ was defined as the highest recorded VO_2_ value when two of three criteria were satisfied: (1) a plateau in VO_2_ peak between two or more workloads, (2) a respiratory exchange ratio >1.00, or (3) a heart rate equivalent to their age predicted maximum (i.e., 220-age). VO_2max_ scores are expressed in units of milliliters per kilogram per minute (ml/kg/min), after controlling for height and weight of the individual.

### Training protocol

#### Aerobic exercise condition

For the aerobic exercise program, a trained exercise leader supervised all sessions. As described by McAuley et al. (2011) and Erickson et al. (2011), participants started by walking for 10 min and increased walking duration weekly by 5-min increments until a duration of 40 min was achieved at week 7. Participants walked for 40 min per session for the remainder of the program. All walking sessions started and ended with approximately 5 min of stretching for the purpose of warming up and cooling down. Participants wore heart rate monitors and were encouraged to walk in their target heart rate zone, which was calculated using the Karvonen method (Strath et al., 2000) according to the resting and maximum heart rates achieved during the baseline maximal graded exercise test. The target heart rate zone was 50–60% of the maximum heart rate reserve for weeks 1–7 and 60–75% for the remainder of the program. Participants in the walking group completed an exercise log at each exercise session. Every 4 weeks, participants received written feedback forms that summarized the data from their logs. Participants with low attendance and/or a low exercise heart rate were encouraged to improve their performance in the following month.

#### Stretching and toning control condition

For the stretching and toning control program, all sessions were led and monitored by trained exercise leaders. All classes started and ended with warm-up and cool-down stretching. During each class, participants engaged in four muscle-toning exercises using dumbbells or resistance bands, two exercises designed to improve balance, one yoga sequence, and one exercise of their choice. To maintain interest, a new group of exercises was introduced every 3 week. During the first weeks, participants focused on becoming familiar with the new exercises, and during the second and third weeks they were encouraged to increase the intensity by using more weight or adding more repetitions. Participants in the stretching and toning control group also completed exercise logs at each exercise session and received monthly feedback forms. They were encouraged to exercise at an approximate intensity of 13–15 on the Borg Rating of Perceived Exertion scale (Borg, 1985) and to attend as many classes as possible.

### Serum BDNF assay

Blood was collected at baseline before the intervention and again immediately after the completion of the program. Blood sampling for BDNF analysis was performed approximately 1 week before the cognitive testing session. Fasted subjects reported to the laboratory at 0600–0800 h, at which time blood from the antecubital vein was collected in serum separator tubes (Becton Dickinson). The blood samples were kept at room temperature for 15 min to allow for clotting, after which the samples were centrifuged at 1100 × g at 4°C for 15 min. Serum was then harvested, aliquoted, and stored at −80°C until analysis. Serum BDNF was quantified using an enzyme-linked immunosorbant assay (Human BDNF Quantikine Immunoassay, DBD00, R&D Systems) according to the manufacturer's instructions.

### BDNF genotyping

We used the BuccalAmp™ DNA Extraction Kit from Epicentre Biotechnologies (Madison, WI USA). Buccal cells were collected from Puritan sterile cheek swabs after rinsing the mouth with tap water. The rs6265 SNP was assayed by a combination of nested PCR and melting-curve analysis with T_m_-shift primers (Wang et al., 2005). The DNA fragment was preamplified from genomic DNA (300 bp) and used as a template for second round (allele-specific) PCR on a Bio-Rad MyiQ thermal cycler, which allows automated melting temperature analysis of the PCR products. One allele-specific primer was designed with a 5′ GC tail, resulting in an easily detectable increase in the melting temperature of the PCR product. For rs6265, the forward and reverse primers used in the first PCR were 5′ ACTCTGGAGAGCGTGAATGG 3′ and 5′ CCAAAGGCACTTGACTACTGA 3′. In the second round PCR, the primer specific to the “G” allele (val) was 5′ GCGGGCAGGGCGGCTCATCCAA CAACTCTTCTAACAC 3′, the primer specific to the “A” allele (met) was 5′ TCATCCAACAACTCTTCTACCAT 3′ and the common primer was 5′ CCAAGGCAGGTTCAAG 3′. Genetic data was analyzed using a dominance model such that Met carriers were combined into a single group.

### Cognitive assessment

The task-switching paradigm is frequently used to assess executive processes (Kramer et al., 1999a; Kray and Lindenberger, 2000; Gratton et al., 2009; Gold et al., 2010; Jimura and Braver, 2010), including cognitive flexibility and inhibition (Verstynen et al., 2012). The task-switch paradigm was administered as part of a larger battery of tasks, but was selected for the present study because (1) it assesses executive functioning and (2) our previous studies have shown that this task is sensitive to exercise interventions (Kramer et al., 1999a,b). Therefore, we selected this task because it met criteria for testing mediation.

**Figure 1:**
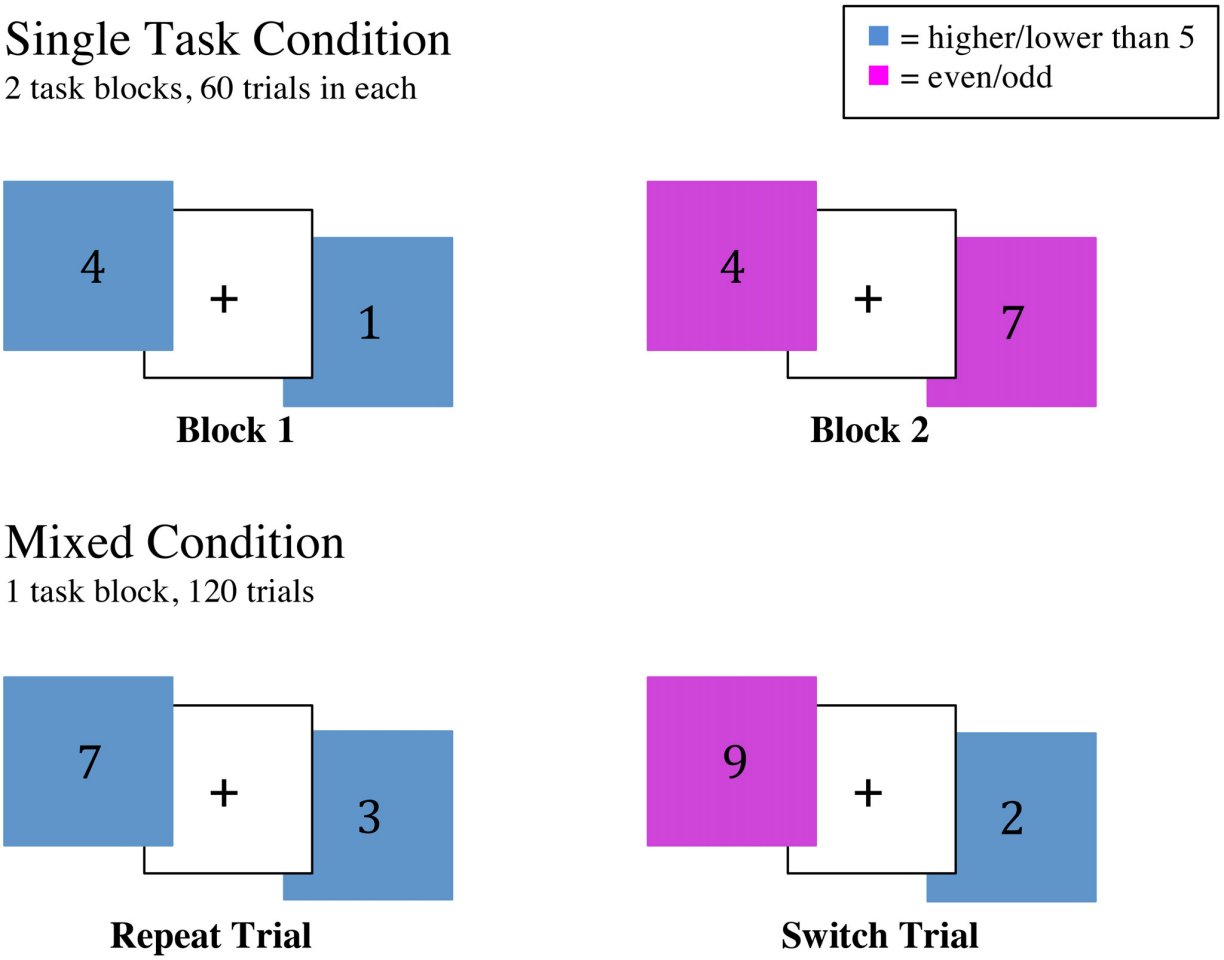
**Display of the task-switch paradigm**. Participants were instructed to respond to a number between 1 and 9 (except for 5) that appeared centrally located on the computer screen. The background color (blue/pink) cued them as to the task they would do (Pink: even/odd; Blue: higher/lower than 5). In the single task condition participants were presented with blocks of a single task whereas in the mixed condition the two conditions were randomly presented.

As described in Voss et al. (2010a), participants utilized color-based cues to determine whether they were to judge whether a number was odd or even, or whether it was low or high (i.e., smaller or larger than 5). The numbers were presented individually for 1500 ms against a pink or blue background. If the background was blue, participants had to determine whether the number was high (“X” key) or low (“Z” key). If the background was pink, participants were to report whether the number was odd (“N” key) or even (“M” key). In both cases, participants were asked to answer as quickly as possible (Voss et al., 2010a). Participants completed a practice block followed by two blocks of individually presented tasks, one block consisted of only the “high or low” task, and the other consisted of only the “odd or even” task. This was followed by a switching block, which included 120 trials with the task in each trial chosen randomly. In this block, some trials were repeated, referred to here as “repeat trials,” and some trials switched between the two conditions, referred to here as “switch trials” (see Figure 1 for task depiction).

Accuracy (% correct) and reaction time (RT) served as indices of performance on the task-switch test. Accuracy (% correct) and time (RT) was recorded separately for single trials, repeat trials within the mixed block, and switch trials within the mixed block. Local switch cost, the difference between repeat trials (when the preceding trial involved the same task) and switch trials (when the preceding trial involved a different task) was calculated for both RT and accuracy (Verstynen et al., 2012). Additionally, global switch cost, the difference between switch trials and single task trials, was calculated for RT and accuracy.

### Statistical analysis

All variables were tested for normality and skew. To assess whether age was inversely associated with serum BDNF, we conducted a multivariable linear regression analysis. *BDNF* genotype was included as a covariate in the model to adjust for effects of the val66met polymorphism on BDNF serum concentrations.

To examine whether the effect of exercise group (walking; stretching) on serum BDNF varied as a function of age, multivariable linear regression was employed using the bootstrap method with 10,000 resampling iterations. Therefore, main effects and interaction effects were estimated from the resampled data repeatedly drawn from the original dataset with replacement. Within the regression model, pre-intervention serum BDNF was included as a covariate to account for any group-level differences in serum BDNF at baseline. Gender and the *BDNF* val66met polymorphism were also adjusted for in the model. Exercise group and age were entered as predictor terms to determine whether age and group independently predicted change in serum BDNF. We also included an age × group interaction in order to test whether age moderated the effect of exercise group on change in serum BDNF.

We also predicted that age would moderate the association between exercise group and cognitive performance post-intervention. To test this hypothesis, we used multivariable linear regression adjusting for gender, years of education, baseline serum BDNF, the *BDNF* val66met polymorphism, and baseline cognitive performance. Thus, rather than modeling within-subject changes in cognitive performance, we assessed group-level differences in performance post-intervention, accounting for individual differences in performance at baseline. Again, bootstrapping was applied to the regression model, using 10,000 iterations. Also entered was group, age as a continuous variable, and their interaction product. A statistical threshold of *p* < 0.05 was used to determine significance for the linear regression analyses. Significant interactions revealed by the linear regression analyses were then subjected to repeated measures analysis of variance tests to assess within-subject changes from pre to post-intervention.

**Figure 2:**
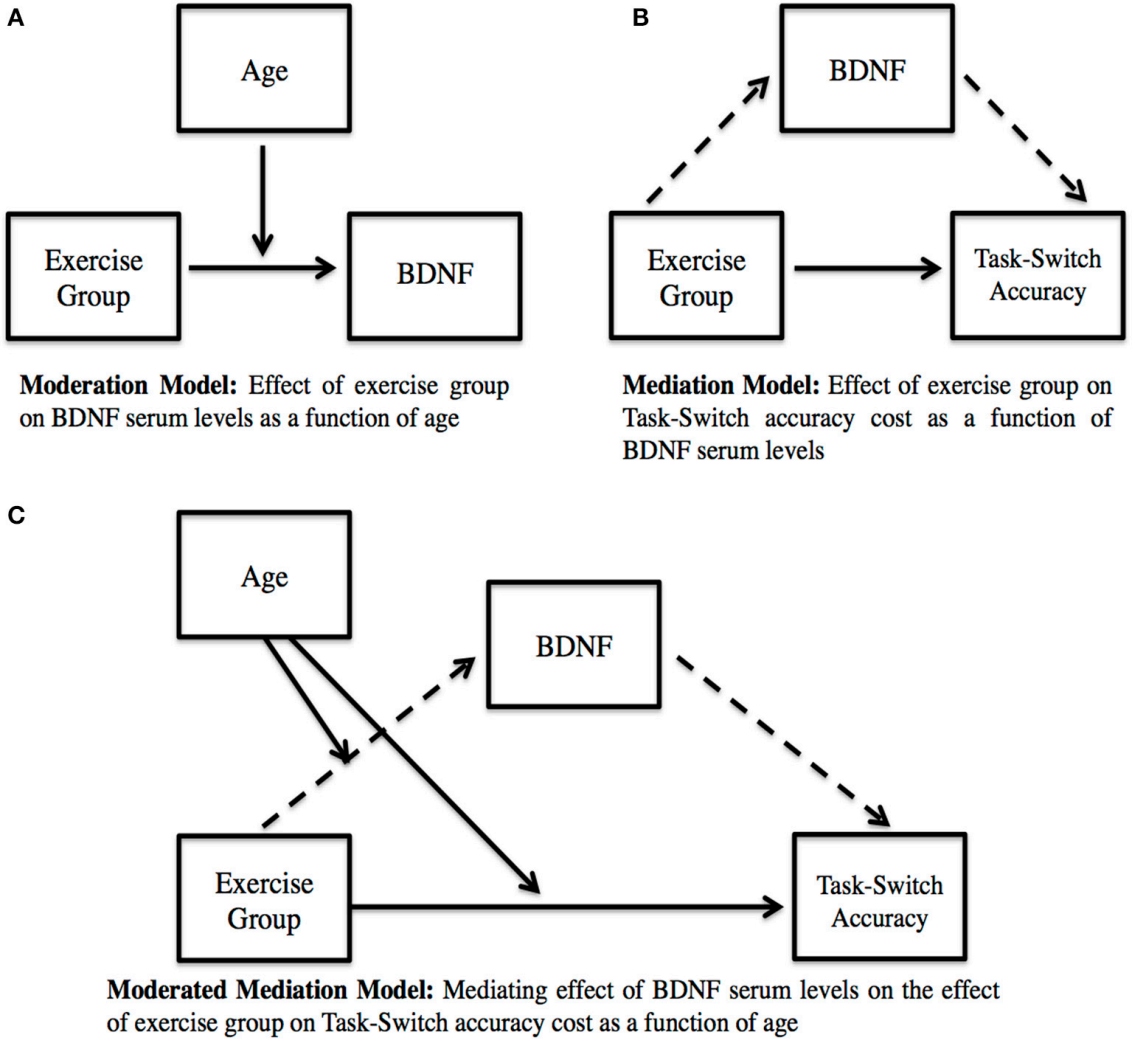
**Moderated mediation model**. **(A)** Moderation Model: Effect of exercise group on BDNF serum levels as a function of age. **(B)** Mediation Model: Effect of exercise group on Task-Switch accuracy cost as a function of BDNF serum levels. Note: Solid arrow indicates direct pathway; Dashed arrow indicates indirect pathway. **(C)** Moderated Mediation Model: Mediating effect of BDNF serum levels on the effect of exercise group on Task-Switch accuracy cost as a function on age. Note: Solid arrow indicates moderation; Dashed arrow indicates mediation.

Finally, conditional process modeling was used to examine the conditional nature by which exercise group predicts cognitive function following the 1-year intervention. Using this procedure, we examined whether BDNF serum levels mediated the relationship between exercise group and cognitive performance, and whether the indirect effect of exercise group on cognitive performance through serum BDNF varied as a function of age. This technique estimates the mediating effect of serum BDNF on the relationship between exercise group and task-switch performance, while accounting for moderating effects of age on (1) the effect of exercise group on serum BDNF and (2) the direct effect of exercise group on task-switch performance. The model format and analyses were conducted using the PROCESS macro by Hayes (2013) in SPSS. This method provided an estimation of both the direct and indirect pathways, resulting in the calculation of 95% confidence intervals (CI) for both direct and indirect effects (see Figure 2 for model depiction). The regression coefficients are displayed in unstandardized form, as the bootstrapped CI's correspond to the unstandardized effects rather than the standardized effects. Mediation results are considered significant if the CI's do not contain 0.

## Results

### Subject characteristics at baseline

The exercise groups did not significantly differ in age, gender, years of education, serum BDNF, or VO_2max_ at baseline (all *p* > 0.05) (see Table 1). Additionally, there were no significant differences between groups on task switch performance indices at baseline (all *p* > 0.05; see Table 2 for task switch accuracy and reaction time data pre and post-intervention).

**Table 2:** **Task-switch performance**.

**Variable**	**Total sample *N* = 90**	**Walking group *N* = 46**	**Control group *N* = 44**
Pre-intervention single accuracy	92.71%	93.13%	92.27%
Post-intervention single accuracy	96.48%	96.47%	96.50%
Pre-intervention repeat accuracy	82.49%	83.33%	81.64%
Post-intervention repeat accuracy	90.17%	92.36%	87.87%
Pre-intervention switch accuracy	77.02%	78.11%	75.91%
Post-intervention switch accuracy	88.86%	91.13%	86.49%
Pre-intervention local accuracy cost	−6.60%	−6.11%	−7.10%
Post-intervention local accuracy cost	−1.60%	−1.48%	−1.73%
Pre-intervention global accuracy cost	−15.69%	−15.02%	−16.36%
Post-intervention global accuracy cost	−7.96%	−5.45%	−10.58%
Pre-intervention single RT (ms)	774.64 (105.35)	774.62 (96.47)	774.67 (114.82)
Post-intervention single RT (ms)	758.97 (106.48)	757.29 (99.94)	760.75 (113.34)
Pre-intervention repeat RT (ms)	994.96 (197.07)	1000.27 (204.64)	989.69 (191.36)
Post-intervention repeat RT (ms)	986.17 (144.69)	991.13 (136.40)	980.88 (154.47)
Pre-intervention switch RT (ms)	1356.34 (194.80)	1358.90 (175.54)	1353.79 (214.32)
Post-intervention switch RT (ms)	1290.29 (239.18)	1298.98 (204.85)	1281 (273.27)
Pre-intervention local RT cost (ms)	361.37 (218.37)	358.63 (225.64)	364.10 (213.37)
Post-intervention local RT cost (ms)	304.12 (184.92)	307.86 (143.17)	300.12 (221)
Pre-intervention global RT cost (ms)	400.86 (163.48)	404.65 (148.15)	397.06 (179.12)
Post-intervention global RT cost (ms)	397.70 (153.57)	407.18 (140.35)	387.58 (167.59)

*Demographic information comparing the two intervention groups: walking intervention, stretching, and toning control*.

*RT, Reaction Time; ms, milliseconds*.

### Age moderates the effect of exercise on change in serum BDNF

The *BDNF* val66met polymorphism was not related to serum BDNF at baseline (*r* = 0.018; *p* = 0.868). Nonetheless, in the regression analyses described below we continued to control for *BDNF* val66met variation because several prior studies have indicated a small, but significant, relation between serum BDNF and the *BDNF* val66met polymorphism (Lang et al., 2009). We also found that females had significantly higher levels of serum BDNF at baseline in this sample (*r* = 0.266; *p* = 0.010) and therefore included gender as a covariate in the analyses below. Consistent with our hypothesis, older ages were associated with lower serum BDNF levels prior to the start of the intervention (*t* = −3.501; *p* = 0.001), after controlling for gender and the *BDNF* val66met polymorphism.

**Figure 3:**
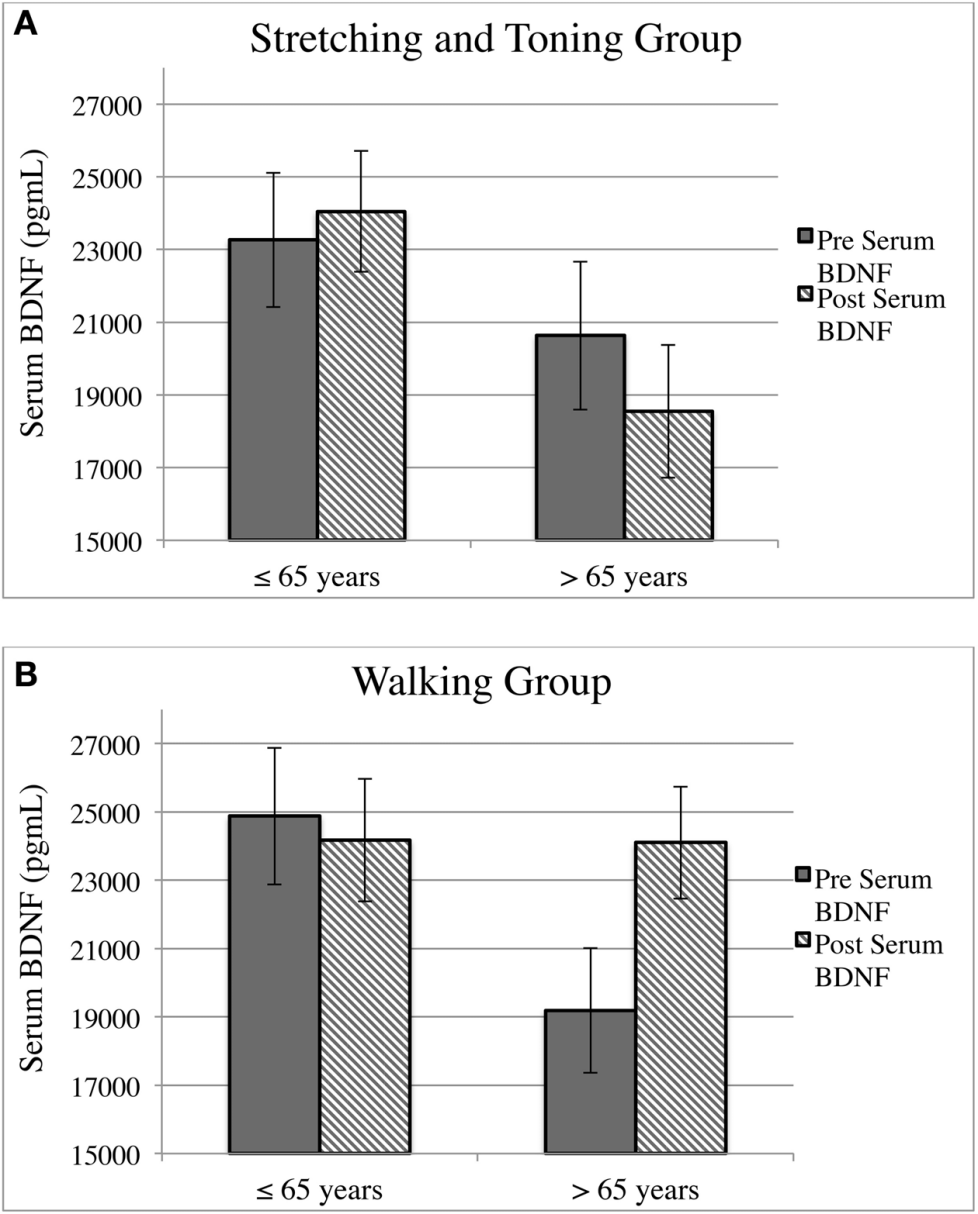
**Moderating effect of age (stratified)**. Note: Effects of exercise on serum BDNF as a function of age. Age stratified using a median split. **(A)** Serum BDNF decreases following the intervention in the control group among adults >65 years. **(B)** Serum BDNF increases following 1 year of walking exercise, specifically for adults aged >65 years.

Collapsed across the intervention and control groups, age did not predict change in serum BDNF over the 1-year period (*B* = −77.69; *p* = 0.489). Similarly, holding age constant, there was not a main effect of exercise group on change in serum BDNF following the intervention, although the association was trending (*B* = 2652.27; *p* = 0.084). However, the lack of main effects was qualified by a significant interaction between age and exercise group on change in serum BDNF (*B* = 471.95; *p* = 0.036) (see Table 3). Specifically, across the 1-year intervention, the stretching and toning group showed a decline in BDNF levels for the oldest individuals. In contrast, there was a positive linear relationship between age and serum BDNF in the walking group, indicating that older aged individuals experienced the greatest increase in BDNF following 1-year of moderate intensity walking exercise (see Figure 3B).

**Table 3:** **Age moderates effect of exercise group on BDNF serum levels at post-intervention**.

	***B***	***SE***	***p***	**LLCI**	**ULCI**
**COVARIATES**					
Baseline serum BDNF	0.473^**^	0.109	0.0001	0.256	0.684
BDNF genotype	1152.29	2055.91	0.560	−2730.59	5415.26
Gender	1137.57	1834.59	0.535	−2555.65	4599.24
**MAIN EFFECTS**					
Age	−77.69	112.27	0.490	−303.22	142.03
Exercise group	2652.27	1472.59	0.078	−373.40	5429.23
**INTERACTION**					
Exercise group × Age	471.95^*^	1.735	0.039	26.261	919.647

*Age as moderator of the effect of exercise group on serum BDNF post-intervention. Coefficients are unstandardized B-values calculated from bootstrap permutation of 10,000 iterations*.

*LLCI, lower-level confidence interval; ULCI, upper-level confidence interval*.

* p < 0.05;

** *p < 0.01*.

For further exploration of this interaction, we used repeated measures analysis of variance and divided the sample into higher and lower aged individuals using the median value for age (Md age = 65). Serum BDNF at baseline and serum BDNF post-intervention were entered as the within subjects factor, age and exercise group were entered as between subjects factors, and *BDNF* genotype and gender were entered as covariates. Consistent with the results from the regression, participation in the walking group offset an age-related reduction in BDNF levels (*F* = 5.45; *p* = 0.022; η^2^_*p*_ = 0.060) (see Figure 3). In particular, 12 months of moderate intensity walking exercise increased serum BDNF levels among adults older than 65 years of age, while similarly aged individuals in the non-aerobic condition showed a decrease in serum BDNF over the 12-month intervention period.

### Age moderates the effect of exercise on task-switch performance

There was not a significant relation between education, baseline serum BDNF, or the *BDNF* val66met polymorphism and performance for any of the task switch outcomes (all *p* > 0.05). However, males had a smaller global accuracy cost at baseline (*r* = −0.210; *p* = 0.046), so gender was used as a covariate in the regression model below. Further, although education and BDNF serum and genotype were not significantly related to task-switch outcomes, we included these variables as covariates in the regression model since prior research has reported effects of education and BDNF on cognitive outcomes in late adulthood (Albert et al., 1995; Erickson et al., 2008; Gunstad et al., 2008). Including these variables in the model provides a more conservative estimate of the associations between exercise group, change in serum BDNF, and change in task-switch performance.

The linear regression model showed that there was not a main effect of group on global accuracy percent cost [(switch trial accuracy-single trial accuracy)/single trial accuracy] post-intervention (*B* = 5.952; *p* = 0.097), although the relation was trending. These results suggest that the control group demonstrated a marginally greater deficit in accuracy between single and switch trials relative to those in the walking group at post-intervention. Additionally, there was not a significant main effect of age on global accuracy cost (*B* = −0.055; *p* = 0.868). But, there was an interaction between age and exercise group on changes in global accuracy cost (*B* = 1.645; *p* = 0.026) (see Table 4). Decomposing this interaction revealed that global accuracy cost remained constant over the 12 month period across all ages within the aerobic exercise group. In contrast, within the stretching and toning control group, there was an age-related increase in percent accuracy cost following the 1-year intervention. Thus, while the magnitude of the global accuracy cost remained constant across all ages in the walking group, older aged individuals within the non-aerobic condition demonstrated a larger cost on performance following the 12-month intervention.

**Table 4:** **Age moderates effect of exercise group on global task switch accuracy cost**.

	***B***	***SE***	***p***	**LLCI**	**ULCI**
**COVARIATES**					
Baseline global accuracy cost	0.186^*^	0.085	0.035	0.039	0.375
Baseline serum BDNF	0.0001	0.0002	0.193	0.000	0.001
BDNF genotype	5.702	5.98	0.339	−4.002	18.473
Gender	3.816	3.515	0.289	−2.659	11.183
Education	2.279	1.297	0.093	−0.249	4.860
**MAIN EFFECTS**					
Age	−0.055	0.328	0.869	−0.717	0.576
Exercise group	5.952	3.420	0.099	−0.694	12.776
**INTERACTION**					
Exercise group × Age	1.645^*^	0.664	0.025	0.382	2.977

*Age as moderator of the effect of exercise group on global accuracy cost post-intervention. Coefficients are unstandardized B-values calculated from bootstrap permutation of 10,000 iterations*.

*LLCI, lower-level confidence interval; ULCI, upper-level confidence interval*.

* *p < 0.05*.

We probed the interaction using a repeated measures analysis of variance, and divided the sample into higher and lower aged individuals using the median age value (Md age = 65). Global accuracy cost at baseline and post-intervention were entered as the within subjects factor, age and exercise group were entered as between subjects factors, and gender, years of education, *BDNF* genotype, and serum BDNF at baseline were included as covariates. In accordance with the results from the regression analysis, age-related reduction in task performance was negated by participation in the aerobic exercise condition (*F* = 5.19; *p* = 0.025; η^2^_*p*_ = 0.064).

In contrast, there was not a significant main effect of exercise group (*B* = 0.602; *p* = 0.633) on local accuracy (switch accuracy − repeat accuracy/repeat accuracy) cost. There was also a non-significant relation between age and local accuracy cost (*B* = 0.156; *p* = 0.103) and a non-significant age × exercise group interaction on local accuracy cost (*B* = 0.265; *p* = 0.136).

Because of the significant effects on global accuracy cost, we conducted *post-hoc* analyses to examine percent accuracy separately for single trials, repeat trials, and switch trials, adjusting for gender, education, trial accuracy at baseline, baseline serum BDNF and the *BDNF* genotype. For single trial accuracy, there was not a main effect of age (*B* = −0.051; *p* = 0.394) or group (*B* = 0.530; *p* = 0.361) nor was there a significant age × group interaction (*B* = −0.092; *p* = 0.433). Similarly, there was not a direct effect of age (*B* = −0.283; *p* = 0.438) or group (*B* = 3.384; *p* = 0.352) on repeat trial accuracy, or an age × group interaction term on repeat trial accuracy (*B* = 0.747; *p* = 0.321). Finally, there was not an effect of age (*B* = −0.075; *p* = 0.832), group (*B* = 4.995; *p* = 0.154), or their product (*B* = 1.126; *p* = 0.124) on switch trial accuracy. These results suggest that the significant age × group interaction on global accuracy cost was specific to global cost, a purer measure of cognitive flexibility, rather than performance on any specific trial type.

Age did not moderate the effect of exercise on RT for any of the variables, including single RT, repeat RT, switch RT, or switch cost (all *p* > 0.05).

### BDNF mediates effect of exercise on task-switch performance as a function of age

The prior analyses demonstrated that the effect of exercise group on BDNF serum levels and task-switch performance varied as a function of age. Specifically, relative to the non-aerobic control condition, 12 months of moderate intensity aerobic exercise led to an increase in serum BDNF and an improvement in task performance (lower cost on global accuracy) that varied with age, with the oldest individuals showing the greatest benefits. Next, we tested whether serum BDNF mediates the association between exercise group and change in global accuracy cost following the 1-year intervention. Additionally, due to the robust age effects observed, a moderated mediation conditional processes model was used to examine whether serum BDNF explained the relation between exercise group and task-switch performance as a function of age. Thus, we first examined whether serum BDNF mediated the relation between exercise group and global accuracy cost. Then, we assessed whether the mediating effect of serum BDNF varied as a function of age. For these analyses, we focused only on the task variables that showed a significant age × group interaction effect (i.e., global accuracy cost). Since gender, education, and the *BDNF* val66met polymorphism did not significantly modify any of the above outcomes, they were removed from subsequent mediation models.

Mediation analyses revealed that there was not a direct effect of exercise group on global accuracy cost, after accounting for baseline serum BDNF and baseline global accuracy cost [*B* = 4.541; *p* = 0.160; CI(SE) −1.831; 10.912 (3.203)]. In contrast, there was a significant indirect effect of exercise group on global accuracy cost through serum BDNF [*B* = 1.317; CI(SE).058; 4.66 (1.007)]. Specifically, participants in the walking group scored, on average, 1.36 percent better on global accuracy cost, which was mediated by exercise-induced changes in serum BDNF levels. The average global accuracy cost was negative (−7.96%) indicating a smaller performance deficit between single and switch trials. Thus, 1 year of moderate-intensity aerobic exercise increased levels of serum BDNF, which, in turn, led to a decrease in cost performance.

**Figure 4:**
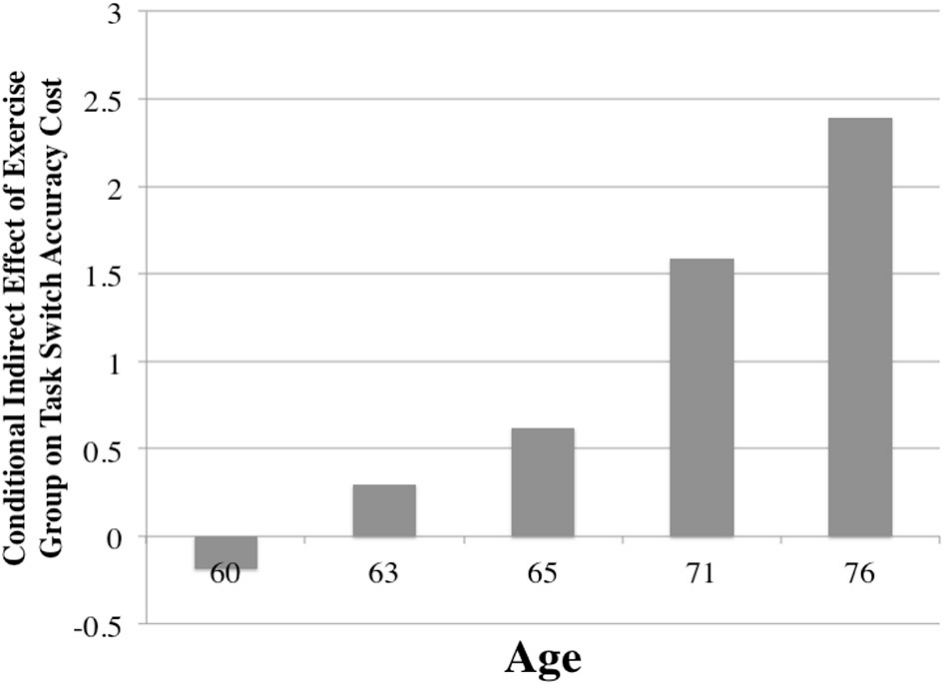
**Mediating effect of BDNF within model, as a function of age**. Note: Moderated mediation conditional indirect effect. Values of the y-axis are the unstandardized mediating effects (coefficients) of BDNF on the relationship between exercise group and task switch accuracy cost. Bars indicate age quintiles (10th, 25th, 50th, 75th, and 90th percentiles) on the x-axis. Sample distribution is as follows: Age <60: *n* = 13; 61–63: *n* = 21; 64–65; *n* = 12; 66–71: *n* = 23; 71–76: *n* = 16; >77: *n* = 7.

Further, the conditional process analysis showed that there was a conditional indirect effect of exercise group on global accuracy cost through BDNF serum that differed by age [*B* = 0.168; CI(SE).009; 0.567 (0.129)] (see Table 5). Further inspection revealed that serum BDNF levels mediated the relationship between exercise group and task-switch performance specifically for older aged individuals (see Figure 4).

**Table 5:** **Moderated mediation model of effects of exercise group on global accuracy cost**.

	***B***	***SE***	***p***	**LLCI**	**ULCI**
**COVARIATES**					
Pre-intervention BDNF	0.0001	0.0002	0.584	−0.0003	0.0005
Baseline global accuracy cost	0.148*	0.062	0.020	0.024	0.271
**MODERATED MEDIATION INDIRECT EFFECT**					
Post-intervention BDNF	0.168^*^	0.129		0.008	0.568
Effect at 10% Age (60 years)	−0.130	0.957		−2.108	1.868
Effect at 25% Age (63 years)	0.373	0.807		−0.761	2.662
Effect at 50% Age (65 years)	0.79	0.799		−0.263	3.162
Effect at 75% Age (71 years)	1.714^*^	1.183		0.260	5.382
Effect at 90% Age (76 years)	2.553^*^	1.716		0.390	7.807

*Moderated mediation model displaying BDNF as mediator of the effect of exercise group on post-intervention global accuracy cost (% correct) as a function of age. Includes overall effect and indirect effect of serum BDNF at different ages (stratified into quintiles)*.

* *p < 0.05—based on confidence interval*.

While the above analyses included age as a continuous predictor, to determine the age at which effects of BDNF mediated task switch accuracy cost, age was divided into quintiles (10th, 25th, 50th, 75th, and 90th percentiles). Effects were then calculated for each of these percentiles of age (60, 63, 65, 71, and 76 years), revealing that serum BDNF mediated the relationship between exercise group and task switch accuracy cost only among participants whose ages were in the 75th [*B* = 1.714; CI(SE) 0.260; 5.382 (1.183)] and 90th percentiles [*B* = 2.553; CI(SE) 0.390; 7.807 (1.716)], or 71 years of age and older. Thus, changes in serum BDNF mediated the relationship between exercise group and task performance, specifically for participants aged 71 years and older.

## Discussion

In line with our hypotheses, changes in serum BDNF mediated the effect of the exercise intervention on task-switch performance. Interestingly, this effect varied as a function of age such that only individuals older than 71 years of age showed a significant mediating effect of BDNF on task-switch performance. Of note, this relationship is significant in a regression model using baseline task-switch performance as a covariate and when examining change in a RM-ANOVA analysis. These results indicate that moderate-intensity physical activity, such as walking, may be more beneficial to both BDNF and task-switching performance for adults over the age of 70 than younger individuals.

Similar to previous research (Ziegenhorn et al., 2007; Flöel et al., 2010), our results demonstrate that older age was associated with lower BDNF serum levels. However, this relationship was not present after the 1-year intervention for the walking exercise group. Exercise increased BDNF levels, but age moderated this relationship such that older adults in the walking group showed increases in BDNF that their younger exercising group members, nor their stretching control counterparts, displayed. This moderating effect of age may account for heterogeneity in the literature that report negligible associations between longer durations of exercise and BDNF levels in middle-aged adults and in samples with a limited age range (Schulz et al., 2004; Schiffer et al., 2009).

Of particular interest, we found evidence that serum BDNF mediated the association between a 12-month exercise intervention and improvements in executive function, but find that this mediating effect is dependent on the age of the sample. Rodent studies have also reported that BDNF mediates the effects of exercise on cognition and related brain pathways (Van Hoomissen et al., 2004; Vaynman et al., 2004; Hopkins and Bucci, 2010; Gómez-Pinilla and Feng, 2012). Yet, the current study is the first to demonstrate this effect in humans through a randomized controlled trial, and including both serum BDNF and *BDNF* genotype. What makes the current analyses unique is that the mediating effect of BDNF on cognition is independent of *BDNF* genotype, and present only in adults over the age of 70. These results highlight that age-related decreases in serum BDNF may be successfully mitigated by participation in exercise and that this might be a critical mechanism by which exercise improves executive function in late adulthood.

Our results also demonstrate that BDNF mediated the effect of exercise on task-switch accuracy cost, but not on response time, in adults aged 70 years or older. Accuracy on the task-switch paradigm is often interpreted as a measure of executive function ability to execute the task demands, while response times are interpreted as a measure of cognitive control, processing speed or efficiency (Hughes et al., 2013). Thus, our results demonstrate that the effects of exercise on the ability to execute the demands of a challenging task that taps into executive functions are mediated by BDNF in adults over the age of 70. We can only speculate as to the reason for the non-significant effects on response times. One possibility is that there are selective effects of BDNF on the neural correlates that support accuracy verses response times (Hughes et al., 2013). Specifically, BDNF may be mediating the effect of exercise on the prefrontal and parietal regions that support executive function (Jimura et al., 2014) and the “top-down” processing required for task-switch accuracy (Phillips et al., 2013), and not the striatum or regions which are associated with processing speed or response time outcomes (Jimura et al., 2014). Still, our results suggest that the modification in the level of serum BDNF is an important pathway by which exercise influences executive function for adults over 70 years of age.

Despite the strengths of our study, there remain several important limitations. First, analyses and interpretation of the results are based on serum BDNF levels, rather than BDNF directly from brain tissue. Importantly, rodent studies have found significant correlations between cortical BDNF and circulating BDNF levels (Karege et al., 2002), despite controversy on the validity of serum BDNF as a proxy for cortical concentrations (Knaepen et al., 2010). Thus, we believe that this is an acceptable limitation given the obvious challenges of obtaining brain tissue from human subjects. Additionally, while the sample size of each intervention group is similar-to or larger-than previous randomized clinical trials, the statistical models employed in the current analyses limit degrees of freedom and require a large sample to detect effects. It is possible that a larger sample would strengthen current findings or reveal effects in younger ages. Additionally, the current sample was mostly female with little representation of racial minorities, preventing generalization to a population outside of female Caucasians. Finally, our sample was relatively homogeneous without outward signs of cognitive impairment. Therefore, a more varied sample with different demographic characteristics and cognitive abilities may find different results than those described here.

The current findings indicate that adults over the age of 70 may gain the most from aerobic exercise in terms of both serum BDNF and task-switch performance. Indeed, the current sample shows no significant difference in BDNF serum levels or task switch performance at follow-up between the control and exercise intervention groups, however when examined as a function of age, BDNF levels significantly mediate the effect of exercise group on improvements in executive function. The results from this study do not preclude positive effects of exercise on other cognitive domains, other age ranges, other populations, or other putative mechanistic pathways (i.e., inflammatory). These findings highlight the importance of considering both age and BDNF when designing exercise interventions and interpreting the mechanism by which exercise improves cognitive performance, particularly in the elderly.

### Conflict of interest statement

The authors declare that the research was conducted in the absence of any commercial or financial relationships that could be construed as a potential conflict of interest.
